# Low occurrence and clonal relatedness of multi-drug resistant *Escherichia coli* carrying transmissible colistin resistance *mcr-1* genes in Ugandan poultry

**DOI:** 10.3389/fvets.2025.1677640

**Published:** 2025-11-17

**Authors:** Martin Wainaina, Dickson Ndoboli, Dreck Ayebare, Irene Mbatidde, Kristina Roesel, Jens Andre Hammerl, Arshnee Moodley, Bernd-Alois Tenhagen, Ulrike Binsker

**Affiliations:** 1 Department Biological Safety, German Federal Institute for Risk Assessment, Berlin, Germany; 2 International Livestock Research Institute, Kampala, Uganda; 3 Department of Veterinary and Animal Sciences, University of Copenhagen, Frederiksberg C, Denmark; 4 Dahlem Research School Biomedical Sciences, Freie Universität Berlin, Berlin, Germany; 5 International Livestock Research Institute, Nairobi, Kenya; 6 Department of Animal Breeding and Husbandry in the Tropics and Subtropics, Universität Hohenheim, Stuttgart, Germany

**Keywords:** poultry production, antimicrobial resistance, Africa, plasmids, bacteria, *mcr-1*, Enterobacterales, ST155

## Abstract

**Introduction:**

Colistin resistance is an emerging global health concern that can lead to limited treatment options for life-threatening human infections. Colistin has widespread use in agriculture in many countries to boost livestock health and productivity. Mobile colistin resistance (*mcr*) genes have been reported globally and facilitate the spread of colistin resistance, but there is limited data on their occurrence in Uganda. This study aimed to identify and characterise *mcr*-carrying *Escherichia coli* from semi-intensive and free-ranging poultry farms in Uganda.

**Methods:**

*mcr*-carrying *E. coli* were isolated and characterised from 402 poultry farms in Wakiso and Soroti districts of Uganda using a combination of selective isolation, PCR detection, antimicrobial susceptibility testing, plasmid transfer assays and next generation sequencing.

**Results:**

Five *E. coli* isolates from five farms (1.2%) were positive for *mcr-1* located on transmissible IncI2(Delta) plasmids of ~63 kb. All isolates had MIC values ranging from 4 to 8 mg/L, belonged to sequence type 155 and exhibited multidrug resistance to antibiotics commonly used on the farms. Whole genome sequencing based phylogeny indicated a close clonal relationship, with SNP distances ranging from 0 to 4 between the isolates from both districts. Lastly, the plasmids were transmissible with a transfer frequency of ~1 × 10^−6^ transconjugants per donor bacteria.

**Conclusion:**

We report *mcr* genes in Ugandan poultry for the first time. Although our study focused solely on poultry farms and revealed a low *mcr* gene occurrence, it highlights the need for attention. Regular One Health monitoring of colistin use and resistance is important to mitigate possible bacterial selection and spread. Policy interventions should focus on promoting the prudent use of antimicrobials in livestock production, and improving biosecurity measures on farms.

## 1 Introduction

Antimicrobial resistance (AMR) is a growing global health concern as treatment options that were once effective against infections no longer are, thereby leading to economic losses (1), and poor health outcomes (2). Antimicrobials are widely used in livestock production in many developing countries for treatment of infections and growth promotion, which contributes to the selection and spread of resistant bacteria (3). Even more concerning is multidrug resistance (MDR), where microorganisms can persist against several structurally unrelated antimicrobials with different modes of action (4). In the event of the transmission from animals to humans through direct contact or the food chain, infections caused by MDR bacteria can be life-threatening, leaving fewer alternatives for therapy (5). Antimicrobial resistance is a leading cause of mortalities in Africa, and requires a One Health approach for detection, response and prevention (6).

Colistin is an important last-line antibiotic of the class polymyxin, used for treating multi-, extensively and pan drug-resistant Gram-negative infections (4, 7). Colistin is also used in several countries to enhance growth and increase production of farm animals (8). Resistance to colistin has long been attributed to mutations in several chromosomal genes (e.g., *pmrAB*, *phoPQ* and *mgrB*) (9). However, the advent of plasmid-mediated mobile colistin resistance (*mcr*) genes gave rise to vast spread of colistin resistance globally (10). To date, ten *mcr* genes (*mcr-1* to *mcr-10*) have been identified, each with various genetic variants (11). *mcr*-genes have been detected in veterinary, human and environmental samples in various African countries, and *mcr*-mediated colistin resistance likely poses a considerable threat to antibiotic therapy (12–16).

Uganda has had an increased demand for and consumption of poultry meat and eggs (17). Analysis of Uganda’s antimicrobial veterinary consumption data from 2021 revealed that imported colistin accounted for 0.1% of total antimicrobial consumption (18). Additionally, several antibiotic formulations containing colistin are registered for veterinary use (19), and colistin-containing multivitamin formulations are commonly used in different poultry production systems (20). There is a paucity of data on the occurrence of *mcr* genes in poultry in Uganda. Few studies have utilized antimicrobial susceptibility testing (AST), plasmid transfer assays, and whole genome sequencing (WGS) for the surveillance of resistant isolates in Uganda. Therefore, we carried out this study to identify and characterise *mcr*-mediated colistin resistance in presumptive *E. coli* originating from poultry farms in Uganda using next generation sequencing and plasmid transfer assays.

## 2 Materials and methods

### 2.1 Sample collection and isolate origin

The study made use of chicken samples from 200 poultry farms in Wakiso district (semi-intensive production) and 202 farms in Soroti district (free-ranging production) in Uganda which were included in a cross-sectional study on antimicrobial resistance. Details on the study design, farm selection, sample collection, and initial sample preparation are reported by Mbatidde et al. (20). In brief, for quantification of colistin-resistant coliforms, one composite fecal sample was collected from one chicken house on each farm. The study used a cross-sectional design, with each farm sampled once. The sample size was calculated using the formula for comparing two proportions and estimated 180 farms per production system for adequate statistical power (21). Sampling was done over a 6-month period in 2021. After enrichment in buffered peptone water, samples were initially grown on MacConkey agar supplemented with 3 mg/L colistin sulphate. One presumptive colistin-resistant *E. coli* was isolated per farm, resulting in 111 presumptive *E. coli* from Soroti samples and 103 *E. coli* from Wakiso samples. The isolates were shipped to the German Federal Institute for Risk Assessment (BfR) in Berlin on nutrient agar slants for further analysis.

### 2.2 Identification of *mcr*-carrying colistin-resistant *E. coli*

At the BfR, bacterial cultures were inoculated in buffered peptone water supplemented with 2 mg/L colistin sulphate (Sigma-Aldrich, Germany) and incubated overnight at 37 °C aerobically while shaking (22, 23). DNA was extracted from 200 μL of the broth using the thermal lysis method, and screened for *mcr-1* to *mcr-9* by conventional multiplex PCRs (24, 25). Cultures that were positive by PCR were plated on ChromID® Colistin R (BioMérieux, France), CHROMagar™ COL-APSE (CHROMagar, France) and MacConkey agar in parallel and incubated at 37 °C overnight to recover pure colistin-resistant Gram-negative bacteria as outlined by Nordhoff et al. (23). Colonies that morphologically resembled *E. coli* were randomly selected (one per sample) and identified using MALDI-TOF mass spectrometry via the direct method using the HCCA matrix for sample preparation (Bruker Daltonics GmbH, Germany). Five *mcr-1*-positive *E. coli* isolates were identified during the screening, originating from five different farms in two districts in Uganda (one isolate from Soroti; four isolates from Wakiso).

### 2.3 Antimicrobial susceptibility testing

*mcr*-positive *E. coli* confirmed by PCR and MALDI-TOF were subjected to AST using standardized broth microdilution assays with 15 antimicrobial agents using a European Union harmonized panel of antimicrobials, defined in Commission Implementing Decision (EU) 2020/1729 (Sensititre™ EU Surveillance *Salmonella*/*E. coli* EUVSEC3 AST Plates, Thermo Scientific, Germany) (26). The following antibiotics and concentration ranges were tested: amikacin (4–128 mg/L), ampicillin (1–32 mg/L), azithromycin (2–64 mg/L), cefotaxime (0.25–4 mg/L), ceftazidime (0.25–8 mg/L), chloramphenicol (8–64 mg/L), ciprofloxacin (0.015–8 mg/L), colistin (1–16 mg/L), gentamicin (0.5–16 mg/L), meropenem (0.03–16 mg/L), nalidixic acid (4–64 mg/L), sulfamethoxazole (8–512 mg/L), tetracycline (2–32 mg/L), tigecycline (0.25–8 mg/L), and trimethoprim (0.25–16 mg/L). Interpretation was done according to epidemiological cut-off (ECOFF) values from the European Committee on Antimicrobial Susceptibility Testing (EUCAST) (22). *E. coli* ATCC 25922 was used as a quality control strain.

### 2.4 Pulsed-field gel electrophoresis (plasmid profiling)

The profiles of extrachromosomal elements in the five isolates were determined by pulsed-field gel electrophoresis (S1-PFGE) using S1-nuclease treated plugs (4 enzyme units/plug) as previously described by Juraschek et al. (27). *Salmonella* serotype Braenderup strain H9812 was used as standard marker for plasmid size determination according to the PulseNet protocol.^1^ PGFE analysis was performed using Bionumerics (v7.6.3; Applied Maths, Belgium). The resulting S1 profiles were analysed by determining fragments sizes >20 kb.

### 2.5 Filter mating assay

The plasmid transfer experiment involved the *mcr*-carrying *E. coli* isolates as donors and *E. coli* strain J53 tolerant to sodium azide (SAZ^R^) as the recipient. All isolates were grown in lysogeny broth (LB) to log phase (OD_600_ of 0.6–0.8). The donor and recipient isolates were gently mixed in a ratio of 1:2 and centrifuged at 3,500 x g for 5 min, after which the supernatant was discarded. The bacterial pellet was resuspended in 100 μL LB, dispensed on 0.22 μm pore-size filter membranes placed on LB agar, and incubated for 24 h at 37 °C. The bacteria were subsequently resuspended in 4 mL LB. A serial dilution was performed on LB agar supplemented with 100 mg/L sodium azide and 2 mg/L colistin sulphate. The resultant transconjugants were tested by *mcr-1* PCR (*mcr1*_320bp_fw 5′-AGTCCGTTTGTTCTTGTGGC-3′/*mcr1*_320bp_rev 5′-AGATCCTTGGTCTCGGCTTG-3′) and AST to prove plasmid transfer using the methods previously detailed (25, 26). Transfer frequency was determined by quantifying the number of transconjugants per donor bacteria.

### 2.6 Whole-genome sequencing

Genomic DNA was isolated from five *E. coli* from overnight broth cultures supplemented with 2 mg/L colistin using the PureLink™ Genomic DNA Kit (Invitrogen, Germany). Sequence libraries were prepared using the Nextera DNA Flex kit (Illumina, United States) utilizing paired-end reads. Short-read sequencing was performed on a NextSeq 500 platform (Illumina) using a 2 × 151 bp cycle configuration [NextSeq 500/550 Mid Output Kit v2.5 (300 Cycles), Illumina]. Long-read sequencing libraries of two *E. coli* isolates were prepared [Rapid Barcoding Kit V14, Oxford Nanopore Technologies (ONT), United Kingdom], and sequencing was performed on an ONT MinION™ Mk1C device using a MinIon Flow Cell (R10.4.1). Base calling that converts sequencing data from signal (.fast5) to sequence format (.fastq) was performed using *Guppy* v3.2.10, and the “fast base calling” option was used to reduce the computational demand.

### 2.7 Bioinformatic analyses

The quality assessment of raw reads included trimming, assembly and quality assessment, taxonomic identification and contamination checks using the AQUAMIS pipeline v1.3.12 (28). Raw long reads were comprehensively analyzed using the *MiLongA* pipeline v1.0.3 (29). This process involved read trimming, quality filtering, and eventually, the creation of hybrid assemblies that combine both the short and long reads using *unicycler* assembler v0.4.8, thereby ensuring highly accurate genome sequences.

*In silico* characterisation of the assemblies was performed using the BakCharak pipeline v3.0.4 (30). This entailed the identification of AMR genes, plasmids incompatibility groups and virulence factors. Typing and classification of the five *E. coli* was performed by determining serotypes, sequence types using the Achtman 4 scheme, Clermont types, and pathotypes (i.e., *stx1*-, *stx2*-, *eae*-, *aat*-, *aggR*-, *aaiC*-, *VT1*-, *VT2*-, *eae*-, *aat*-, *aggR-* and *aaiC-*positive *E. coli*). The average nucleotide identity (ANI) scores that ascertain taxonomy were determined using *pyani* v0.2.12 (31). Lastly, the relatedness of the isolates was determined using single nucleotide polymorphisms (SNPs) shared among isolates, and phylogenetic trees were generated using CSI Phylogeny v1.4 (32). Annotation of the hybrid assembly was conducted on the PATRIC bioinformatics resource center (33, 34). The *mcr*-bearing plasmids were visualised using the hybrid assemblies via *Accelrys Gene* v2.5, and plots were created on R statistical software environment v4.3.2 (35). The similarity of *mcr*-bearing plasmids in the study isolates, and their homology with other plasmids globally was analyzed using CSI Phylogeny and *blastn* (against the core genome nucleotide database) searches, respectively.

## 3 Results

### 3.1 Phenotypic resistance

Multiplex-PCR for *mcr-1* to *mcr-9* of presumptive isolates cultivated in colistin-supplemented broth yielded five confirmed *mcr*-*1*-carrying *E. coli*, four originating from the semi-intensive farms in Wakiso (4/103, 3.9%) and one from a free-ranging farm in Soroti district (1/111, 0.9%). Phenotypic AST revealed that the *E. coli* had minimum inhibitory concentration [MIC] values of 4–8 mg/L to colistin. Additionally, resistance to ampicillin, ciprofloxacin, gentamicin, sulfamethoxazole, tetracycline and trimethoprim was observed (Table 1). Lastly, three isolates were resistant to nalidixic acid. Susceptibility to amikacin, azithromycin, chloramphenicol, cefotaxime, meropenem, ceftazidime, and tigecycline was observed in all strains.

**Table 1:** Susceptibility of *mcr*-carrying *E. coli* isolates from poultry farms in Uganda to 15 antibiotics.

Isolate	AMK	AMP	AZI	CHL	CIP	COL	FOT	GEN	MERO	NAL	SMX	TAZ	TET	TGC	TMP
*E. coli* EUCAST ECOFF values	16	16	32	32	0.12	4	0.5	4	0.25	16	128	1	16	1	4
23-MO00583-5	≤4	>32	8	≤8	0.5	4	≤0.25	>16	≤0.03	16	>512	0.5	>32	0.5	>16
23-MO00719-1	≤4	>32	4	≤8	0.5	8	≤0.25	>16	≤0.03	8	>512	≤0.25	>32	≤0.25	>16
23-MO00728-1	≤4	>32	4	≤8	0.5	4	≤0.25	>16	≤0.03	16	>512	≤0.25	>32	≤0.25	>16
23-MO00748-1	≤4	>32	4	≤8	1	8	≤0.25	>16	≤0.03	8	>512	≤0.25	>32	≤0.25	>16
23-MO00770-1	≤4	>32	4	≤8	0.5	8	≤0.25	>16	≤0.03	8	>512	≤0.25	>32	≤0.25	>16

Shaded cells indicate resistance and MIC values are given in mg/L. AMK, amikacin; AMP, ampicillin; AZI, azithromycin; CHL, chloramphenicol; CIP, ciprofloxacin; COL, colistin; FOT, cefotaxime; GEN, gentamicin; MERO, meropenem; NAL, nalidixic acid; SMX, sulfamethoxazole; TAZ, ceftazidime; TET, tetracycline; TGC, tigecycline; TMP, trimethoprim; ECOFF, epidemiological cut-off values; European Committee on Antimicrobial Susceptibility Testing, EUCAST.

### 3.2 Next generation sequencing characterisation

The five isolates were further confirmed as *E. coli* with a median ANI score of 96.1 when compared with *E. coli* O157:H7 str. Sakai reference genome (accession no. GCA_000008865.2). The genome size varied between 5.015 and 5.023 Mb (Supplementary Table 1). *In silico* multilocus sequence typing (MLST) revealed that all isolates had identical allelic profiles [*adk* (6); *fumC* (4); *gyrB* (14); *icd* (16); *mdh* (24); *purA* (8); *recA* (14)], which denote sequence type 155 (ST 155). Additionally, all isolates were serotype O133:H25, Clermont type B1, but did not resemble any of the predefined *E. coli* pathotypes (STEC, EHEC, etc.). The genome assemblies have been deposited in GenBank under BioProject PRJNA1181821 (accession numbers GCA_053044225.1-GCA_053044365.1, BioSamples SAMN44571232-SAMN44571236).

**Figure 1:**
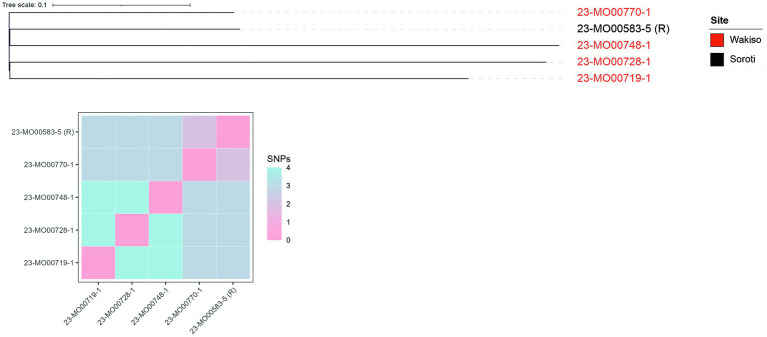
Phylogenetic relationship of the genomes of colistin-resistant *E. coli* isolates recovered from poultry farms in Wakiso and Soroti based on the concatenated alignment of single nucleotide polymorphisms (SNPs). A hybrid assembly (both long- and short-read sequencing approaches) of isolate 23-MO00583-5 was utilized as the reference (R). The number of SNPs used in the analyses are illustrated in the heat map based on 96.7% similarity of all isolates to the reference sequence. The few SNPs shared among isolates suggest a clonal relationship.

Interestingly, phylogenetic analyses revealed that the five isolates from both production systems shared between 0 and 4 SNPs, suggesting a strong clonal relationship (Figure 1). This is despite Wakiso and Soroti districts being more than 300 km apart by road, and sampling occurring more than 4 months apart. All isolates also showed 96.7% similarity to the reference hybrid assembly, further confirming the close genetic relatedness of the isolates.

The short read WGS data showed 13 AMR genes using the AMRFinder database. All isolates carried *mcr-1.1* responsible for colistin resistance. Additional acquired genes found in all isolates were *aac(3)-IId*, *aph(6)-Id*, *blaEC*, *bla*_TEM-1_, *dfrA14*, *qnrS1*, *sul3*, and *tet*(A), which can confer resistance to several antibiotics, including fosfomycin, gentamicin, quinolones, streptomycin, sulfonamides, tetracycline, and trimethoprim (Supplementary Table 2). Furthermore, the isolates harbored efflux-related genes (*acrF* and *emrD*), and had alteration of chromosomal genes associated with the development of resistance (*glpT_E448K*, *mdtM*). One isolate exhibited a *pmrB_A159V* mutation, suggested to be involved in colistin resistance (36).

All five isolates carried a total of 83 virulence genes each, which were in the broad virulence categories of: adherence (21 genes), antimicrobial activity/competitive advantage (1), effector delivery system (32), immune modulation (1), invasion (4), nutritional/metabolic factor (20), and regulation (4) (Supplementary Table 3). Examples of the genes in these categories are the adherence-associated *fim* operon, efflux-related *acrB*, a complete type VI secretion system, immune modulation *gndA* gene, invasion-associated *ibe* genes, and a complete siderophore system (*iro)* necessary for iron utilization.

### 3.3 Plasmid characteristics

**Figure 2:**
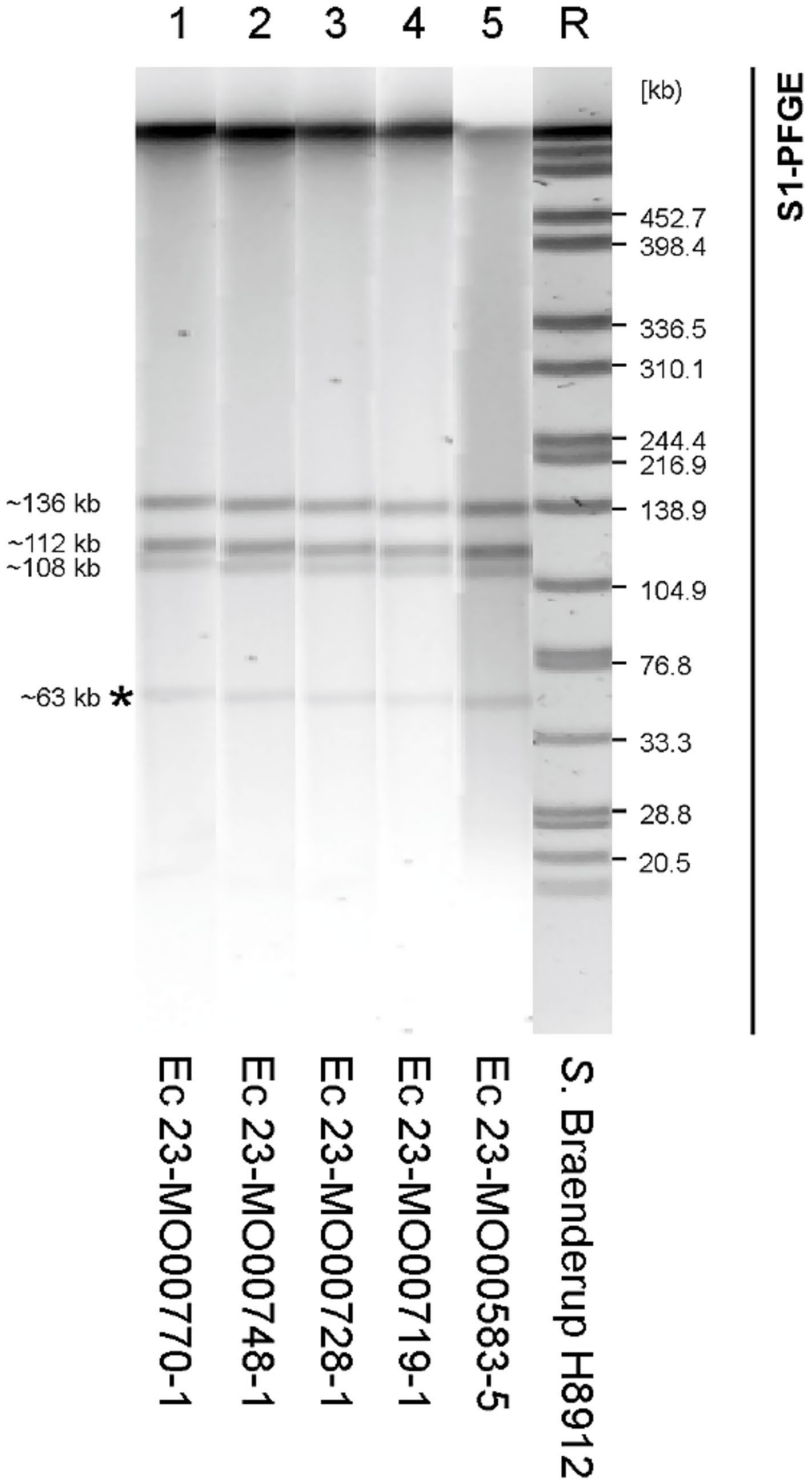
Pulsed-field gel electrophoresis (PFGE) image showing bands at the expected ~63 kb region, confirming the conserved structure and size of plasmids in the study isolates. R, reference strain (*Salmonella* serovar Braenderup H9812); Ec, *Escherichia coli.*

The determination of the plasmid profiles by S1-PFGE confirmed the strong genetic relationship of the isolates also at extrachromosomal level. All isolates exhibited four bands of similar sizes covering genomes of 33–140 kb (Figure 2). Based on the close relationship of all five *E. coli* isolates, two isolates (23-MO00583-5 from Soroti; 23-MO00719-1 from Wakiso), representing both production systems, were selected for the hybrid assembly using both short and long read sequencing approaches and used as a reference genome in the study.

**Figure 3:**
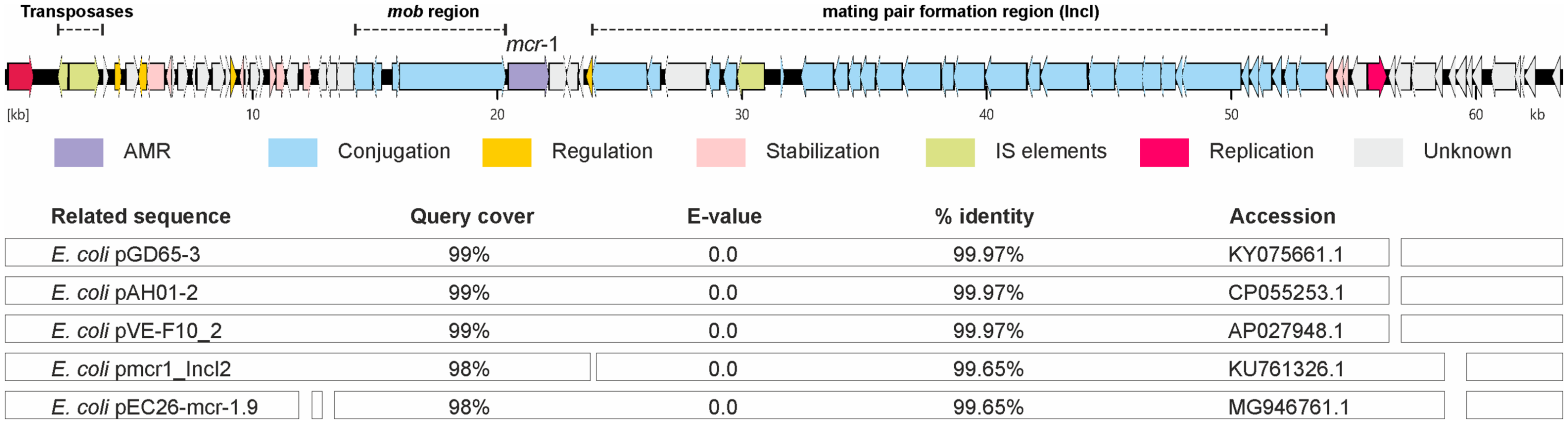
A linear, true to scale illustration of the circular *mcr*-carrying IncI2(Delta) plasmid from *E. coli* isolate 23-MO00583-5 (p583-5) highlighting the *mcr-1.1* gene and its association with genes related to plasmid transfer and pilus assembly. The leading *blastn* search results showing homology of p583-5 to others with diverse country and host sources are also presented.

Hybrid assembly and sequence comparisons revealed five plasmid incompatibility (Inc) types in all isolates: IncI2(Delta), IncFII(pRSB107), IncFIB, IncFII(pCoo), and IncX1, but the *mcr-1.1* genes were only found on IncI2(Delta) plasmids. The *in-silico* analyses showed that *mcr-1.1* was located on a 63,569 bp IncI2(Delta) plasmid (p583-5) that did not harbour other AMR or virulence genes, but encoded transfer genes (*traL*, *traE* and *traD*) as well as pilus-related proteins (PilV, PilP, and PilM) (Figure 3). The IS*605* insertion element was found ≥18 kb upstream of *mcr-1*, and its role in *mcr* mobility is therefore uncertain (Supplementary Table 5). Comparison of the p583-5 sequence with publicly available sequences on NCBI’s *blastn* database revealed a close similarity to plasmids from other *E. coli,* mainly originating from Asia, which were isolated from sources such as chicken (CP055253.1) and human blood (KU761326.1) (Figure 3). Additionally, p583-5 resembled homologues of plasmids from clinically relevant bacteria such as *Cronobacter* and *Salmonella*, which contained a homologue of p583-5 incorporated into a large hybrid plasmid (Supplementary Table 4). Lastly, the *mcr*-carrying plasmids from the other isolates in the study were 99.8% identical to p583-5 (Accession number PQ659175), differing in only one SNP, proving it to be an appropriate representative of the other four isolates in the study. The filter mating assays confirmed the transmissibility of the *mcr*-bearing plasmids from all five *E. coli* donors using *E. coli* J53 as the recipient, with a transfer frequency of ~1 × 10^−6^ (range 10^−5^–10^−7^) transconjugants per donor bacteria (i.e., approximately one recipient bacterium becomes a transconjugant from every million donor bacteria). Both *mcr-1* and phenotypic colistin resistance were confirmed in the transconjugants (Table 2).

**Table 2:** Representative data of susceptibility testing of *mcr*-carrying 23-MO00583-5 donor, recipient and transconjugant, showing the acquisition of colistin resistance.

Isolate (plasmid)	Description	AMK	AMP	AZI	CHL	CIP	COL	FOT	GEN	MERO	NAL	SMX	TAZ	TET	TGC	TMP
*E. coli* J53	Recipient	≤4	≤4	≤2	≤8	0.03	≤1	≤0.25	≤0.5	≤0.03	≤4	≤8	≤0.25	≤2	≤0.25	≤0.25
23-MO00583-5	Donor	≤4	>32	8	≤8	0.5	4	≤0.25	>16	≤0.03	16	>512	0.5	>32	0.5	>16
*E. coli* J53 (p583-5-T1)	Transconjugant	≤4	≤4	≤2	≤8	0.06	4	≤0.25	≤0.5	≤0.03	≤4	≤8	≤0.25	≤2	≤0.25	≤0.25

Shaded cells indicate resistance (MIC values in mg/L). AMK, amikacin; AMP, ampicillin; AZI, azithromycin; CHL, chloramphenicol; CIP, ciprofloxacin; COL, colistin; FOT, cefotaxime; GEN, gentamicin; MERO, meropenem; NAL, nalidixic acid; SMX, sulfamethoxazole; TAZ, ceftazidime; TET, tetracycline; TGC, tigecycline; TMP, trimethoprim.

## 4 Discussion

We report *mcr*-mediated colistin resistance in poultry from Uganda for the first time. Despite finding few colistin-resistant *E. coli* in the investigated poultry farms (1.2%), the transmissibility of *mcr*-bearing plasmids highlights the importance of measures to prevent the selection and future spread of colistin resistance in the country.

Whole genome sequencing revealed the presence of several resistance and virulence genes. The isolates were not assigned to any specific *E. coli* pathotypes. However, various identified virulence factors could play a role in biofilm formation (e.g., *csgA*, *csgB*, *fimH*) (37), and survival in harsh or nutrient-deficient environments (e.g., *entA*, *entB, iro*). These features give the isolates competitive advantage and enable persistence in unconducive environments. Additionally, a mutation in the *pmrB* gene which is thought to mediate colistin resistance was found (38). The bacteria belonged to ST155 which has been associated with urinary tract infections in humans in the country, and ill chickens in South Africa (13, 39). Additionally, there is the potential for spread of resistant bacteria within the farms if manure from the affected chicken coops is used for crop production. However, the colonisation, persistence and zoonotic potential of these bacteria would require further investigation using *in vitro* and *in vivo* models, which was beyond the scope of this study. All five isolates had *mcr* genes located on IncI2 plasmids. This plasmid type has previously been associated with *mcr-1* genes (40). The associated plasmids in all isolates showed low transfer frequencies, suggesting a minimal risk of transfer under natural conditions. Furthermore, given the many factors that can influence plasmid transfer *in vivo* (41), significant transfer frequencies cannot be ruled out.

Resistance genes to several other important antibiotics were found and phenotypic testing confirmed multidrug resistance of the isolates. MDR bacteria can lead to economic losses in animal production and increased veterinary costs from treatment failures. Their zoonotic potential can lead to high morbidity and mortality rates in humans, especially in vulnerable populations (e.g., neonates, the elderly and immunocompromised persons) (2). Frequent use of these antibiotics without veterinary oversight has been reported for the treatment and prevention of infections of poultry in the visited farms in Soroti and Wakiso districts (20). This practice exerts selective pressure, potentially driving the emergence and spread of MDR isolates, particularly in *E. coli*, which are well known for their genetic adaptability (42).

Colistin resistance in Uganda has only recently been reported in hospital settings from *mcr*-negative *Citrobacter freundii* (43) and *Klebsiella pneumoniae* (44, 45). Colistin is a reserve-drug for treating infections with MDR Gram-negative bacteria in humans (46). However, colistin-resistant phenotypes and genotypes are now reported in Africa from bacteria of livestock origin (47, 48). In Uganda, colistin is registered for use in poultry, and imported colistin accounts for 0.1% of total veterinary antimicrobial consumption (18, 19). Colistin-containing formulations, often also registered as non-antibiotic products, are sold over the counter for veterinary use without professional supervision in several African countries, including Uganda (18, 19, 49, 50). Consequently, colistin resistance has been observed on the continent in human and animal populations (49), including in naïve populations such as neonates (51) and wildlife (49). Restricting colistin use in livestock production could help prevent the emergence and limit the spread of colistin resistance. For example, China reduced colistin-resistant *E. coli* in poultry after implementing restrictions on non-therapeutic colistin use in 2017, following the discovery of plasmid-mediated colistin resistance genes in the country (10, 52). Likewise, a decrease in colistin-resistant commensal *E. coli* from chicken carcasses was observed in South Africa after introduction of tighter regulations on veterinary colistin usage (53). A multi-sectoral and multi-disciplinary approach involving stakeholder dialogue that includes farmers, veterinary extension officers and agrovet stores (i.e., farmer supply vendors) is essential in addressing any restriction of colistin use in agriculture in Uganda and to explore potential alternatives. This One Health approach has been taken in other countries such as South Africa (54), and could help safeguard colistin as a critical treatment option for MDR Gram-negative infections (7). Developing country-specific solutions that enhance livestock productivity, promote food security and protect livelihoods, while minimising the risk of colistin resistance, is essential (55, 56). Such solutions should be informed by comprehensive risk assessment and cost–benefit analyses to ensure balanced and sustainable policies.

A strong clonal relationship was found between the isolates as they shared a maximum of four SNPs despite being from different farms, production systems and regions. Dissemination of *mcr*-containing bacteria over wide distances could occur through human and animal movement (e.g., supply of chicks), vehicles, or shared farm inputs (e.g., animal feeds). Regional spread can also occur via common water sources, equipment, manure, and personnel (e.g., veterinarians and farm workers), as well as other living vectors such as small mammals, migratory/free-ranging birds, and flies (55). The sale and consumption of contaminated poultry products may further support spread of *mcr* genes within and beyond the poultry sector (55). The potential of clonal spread combined with plasmid mobility could facilitate the rapid dissemination of colistin resistance. This underscores the value of good farm management practices, biosecurity measures and the prudent use of antimicrobials.

Our study only considered chicken farms and found few resistance isolates. However, comprehensive studies that involve the sampling of the environment, livestock, synanthropic wildlife and humans are needed to understand the prevalence of these resistance genes and the transmission dynamics of mobile colistin resistance in a One Health space.

An integrated AMR surveillance in Uganda is vital for regular monitoring and to inform measures that prevent clonal spread. This has been acknowledged by the key government ministries through the jointly-endorsed One Health Strategic Plan (2018–2022), and the new National Action Plan on AMR 2024–2029 (57, 58). Uganda has committed to building an effective AMR surveillance system supported by the government and donors such as the Fleming Fund. The system aims to establish a network of human and animal surveillance sites and laboratories to test bacteria from patients, animals and the environment (59). To ensure consistent and comparable colistin resistance data, the adoption of broth microdilution methods is necessary as disk diffusion is not recommended for detecting colistin resistance (60). Monitoring colistin usage and resistance will contribute to assessing national, regional and global resistance trends across medical, agricultural and food sectors (61–63). The similarity of the *mcr*-bearing plasmids in this study to those originating from Asian countries highlights the importance of this international collaboration in monitoring and managing mobile colistin resistance.

Our study had a few limitations. We investigated only one isolate per farm which may have underestimated the occurrence of these genes on farms. A selective concentration of 3 mg/L of colistin was used during field sample preparation, which likely excluded isolates with lower MIC values and favoured the growth of highly colistin-resistant isolates such as those found in this study. Future surveillance studies determining MIC values across a range (e.g., 1–16 mg/L as done in this study) using the broth microdilution method, and applying EUCAST breakpoints may provide a more comprehensive view of the prevalence of colistin (60). Molecular testing of isolates via PCR or WGS can further strengthen surveillance by determining *mcr* genes even in Enterobacterales isolates with MIC ≤2 mg/L, thus tracking the “silent” dissemination of *mcr* genes in phenotypically susceptible isolates (64). Additionally, investigating resistance to other antibiotics using WGS, including the role of chromosomal colistin-resistance genes, would provide a better understanding of broader resistance profiles and inform targeted control strategies.

In conclusion, a low occurrence of closely related colistin-resistant isolates with transmissible *mcr*-carrying plasmids was found. Our study offers preliminary evidence of colistin resistance in Uganda. Robust monitoring of colistin usage and resistance is required to ensure that an emerging trend is not missed. Lastly, studies in other livestock sectors, in animal derived foods, and in the medical field are recommended to understand the epidemiological landscape of resistance genes and transmission dynamics within and between human and animal hosts. This is crucial to creating more targeted and effective control measures.

## Data Availability

The original contributions presented in the study are publicly available. This data can be found here: NCBI, Bioproject with accession number PRJNA1181821.

## Footnotes

^1^ https://pulsenetinternational.org/protocols/pfge/
