# Supplementary material for: Low occurrence and clonal relatedness of multi-drug resistant Escherichia coli carrying transmissible colistin resistance mcr-1 genes in Ugandan poultry
Source: Front Vet Sci. 2025 Nov 17;12:1677640. doi: 10.3389/fvets.2025.1677640 (PMC12667025; doi:10.3389/fvets.2025.1677640)
Supplement: Supplementary file 1 [file Supplementary_file_1.zip › Supplementary Tables/Supplementary Tables 1-4.docx]

**Low occurrence and clonal relatedness of multi-drug resistant *Escherichia coli* carrying transmissible colistin resistance *mcr-1* genes in Ugandan poultry**

Martin Wainaina , Dickson Ndoboli , Dreck Ayebare , Irene Mbatidde , Kristina Roesel , Jens Andre Hammerl , Arshnee Moodley , Bernd-Alois Tenhagen , Ulrike Binsker

# **Supplementary information**

Supplementary Table 1: A summary of whole genome sequencing and assembly statistics

| Sample Name | No. Reads | Q30 Base Fraction | Coverage Depth | No. Contigs Length (1000) | N50 | Total Length | GC | Reference Accession | Reference Coverage | Reference Length | Reference Similarity | Contamination status |
| --- | --- | --- | --- | --- | --- | --- | --- | --- | --- | --- | --- | --- |
| 23-MO00583-5 | 3974663 | 0.918 | 58.9 | 109 | 148104 | 5018554 | 50.6 | NZ_CP012380.1 | 0.925 | 4847481 | 0.829 | FALSE |
| 23-MO00719-1 | 4625749 | 0.9167 | 68.6 | 112 | 148104 | 5017680 | 50.6 | NZ_CP012380.1 | 0.9246 | 4847481 | 0.829 | FALSE |
| 23-MO00728-1 | 4001291 | 0.9172 | 59.4 | 107 | 148104 | 5023140 | 50.61 | NZ_CP012380.1 | 0.9252 | 4847481 | 0.829 | FALSE |
| 23-MO00748-1 | 4242964 | 0.9152 | 62.8 | 112 | 123136 | 5015568 | 50.6 | NZ_CP012380.1 | 0.9247 | 4847481 | 0.829 | FALSE |
| 23-MO00770-1 | 4081655 | 0.917 | 60.4 | 103 | 148713 | 5019883 | 50.6 | NZ_CP012380.1 | 0.9249 | 4847481 | 0.829 | FALSE |

Supplementary Table 2: A summary of antimicrobial resistance genes identified using ResFinder database on whole genome sequences of phenotypically colistin-resistant isolates with mcr genes.

| **Resistance gene** | **Antibiotic subclass** | **Predicted product** | **Isolate ID** |
| --- | --- | --- | --- |
| *aac(3)-IId* | Gentamicin | Aminoglycoside N-acetyltransferase AAC(3)-IId | 23-MO00583-5, 23-MO00719-1, 23-MO00728-1, 23-MO00748-1, 23-MO00770-1 |
| *acrF* | Efflux | Multidrug efflux RND transporter permease subunit AcrF | 23-MO00583-5, 23-MO00719-1, 23-MO00728-1, 23-MO00748-1, 23-MO00770-1 |
| *aph(6)-Id* | Streptomycin | Aminoglycoside O-phosphotransferase APH(6)-Id | 23-MO00583-5, 23-MO00719-1, 23-MO00728-1, 23-MO00748-1, 23-MO00770-1 |
| *blaEC* | Beta-lactam | BlaEC family class C beta-lactamase | 23-MO00583-5, 23-MO00719-1, 23-MO00728-1, 23-MO00748-1, 23-MO00770-1 |
| *bla*_TEM-1_ | Beta-lactam | Broad-spectrum class A beta-lactamase TEM-1 | 23-MO00583-5, 23-MO00719-1, 23-MO00728-1, 23-MO00748-1, 23-MO00770-1 |
| *dfrA14* | Trimethoprim | Trimethoprim-resistant dihydrofolate reductase DfrA14 | 23-MO00583-5, 23-MO00719-1, 23-MO00728-1, 23-MO00748-1, 23-MO00770-1 |
| *glpT_E448K* | Fosfomycin | *Escherichia* fosfomycin resistant GlpT | 23-MO00583-5, 23-MO00719-1, 23-MO00728-1, 23-MO00748-1, 23-MO00770-1 |
| *mcr-1.1* | Colistin | Phosphoethanolamine--lipid A transferase MCR-1.1 | 23-MO00583-5, 23-MO00719-1, 23-MO00728-1, 23-MO00748-1, 23-MO00770-1 |
| *mdtM* | Efflux | Multidrug efflux MFS transporter MdtM | 23-MO00583-5, 23-MO00719-1, 23-MO00728-1, 23-MO00748-1, 23-MO00770-1 |
| *pmrB_A159V* | Colistin | *Escherichia* colistin resistant PmrB | 23-MO00719-1 |
| *pmrB_Y358N* | Colistin | *Escherichia* colistin resistant PmrB | 23-MO00583-5, 23-MO00719-1, 23-MO00728-1, 23-MO00748-1, 23-MO00770-1 |
| *qnrS1* | Quinolone | Quinolone resistance pentapeptide repeat protein QnrS1 | 23-MO00583-5, 23-MO00719-1, 23-MO00728-1, 23-MO00748-1, 23-MO00770-1 |
| *sul3* | Sulfonamide | Sulfonamide-resistant dihydropteroate synthase Sul3 | 23-MO00583-5, 23-MO00719-1, 23-MO00728-1, 23-MO00748-1, 23-MO00770-1 |
| *tet*(A) | Tetracycline | Tetracycline efflux MFS transporter Tet(A) | 23-MO00583-5, 23-MO00719-1, 23-MO00728-1, 23-MO00748-1, 23-MO00770-1 |

Supplementary Table 3: A summary of virulence factors identified by utilising the virulence factor database (http://www.mgc.ac.cn/VFs/main.htm).

| **Virulence family** | **Virulence gene(s)** | **Isolate ID** |
| --- | --- | --- |
| Adherence | *cfaA, cfaB, cfaC, cfaD, cgsD, cgsE, cgsF, cgsG, csgA, csgB, csgC, fdeC, fimF, fimG, fimH, yagV, yagW, yagX, yagY, yagZ, ykgK* | All (23-MO00583-5, 23-MO00719-1, 23-MO00728-1, 23-MO00748-1, 23-MO00770-1) |
| Antimicrobial activity/Competitive advantage | *acrB* | All |
| Effector delivery system | *clpV, espL1, espR1, espX1, espX4, espX5, fha, gspC, gspD, gspE, gspF, gspG, gspH, gspI, gspJ, gspK, gspL, gspM, hcp1, hcp2, tssA, tssB, tssC, tssF, tssG, tssJ, tssK, tssL, tssM, vgrG* | All |
| Immune modulation | *gndA* | All |
| Invasion | *AAA92657, ibeB, ibeC, ompA* | All |
| Nutritional/Metabolic factor | *allB, entA, entB, entC, entD, entE, entF, entS, fepA, fepB, fepC, fepD, fepE, fepG, fes, iroB, iroC, iroD, iroE, iroN* | All |
| Regulation | *fur, phoP, rcsB, rpoS* | All |

Supplementary Table 4: Homology of p583-5 to plasmids from other Enterobacterales except *E. coli*.

|  | **KX505142.1** | **MH522426** |
| --- | --- | --- |
| **Parameter** |  |  |
| Size (bp) | 65,203 | 277,473 |
| Mobile genetic element finder | | |
| Inc type (Identity %) | IncI2 (100.00), IncHI2 (100.00), IncHI2A (99.52) | IncI2 (100.00) |
| Resistance genes | *mcr-1.1* (2x), *fosA*3, *bla*_CTX-M-14_, *sul1*, *bleO* | *mcr-1.1* |
| Nucleotide blast | | |
| Max score | 47,655 | 43,655 |
| Total score | 1.192e+05 | 1.137e+05 |
| Query coverage (%) | 99.00 | 91.00 |
| E-value | 0.0 | 0.0 |
| Identity (%) | 99.42 | 99.81 |
| Microorganism | *Cronobacter* *sakazakii* | *Salmonella* *enterica* subsp. *enterica* serovar Typhimurium |
| Sample type/host | Chicken faeces | *Homo* *sapiens* |

Supplementary Table 5: Annotation of plasmid p583-5 from isolate 23-MO00583-5 using the PATRIC bioinformatic resource centre.

Table 2.xlsx
